# Brown adipose tissue: is it affected by intermittent hypoxia?

**DOI:** 10.1186/1476-511X-9-121

**Published:** 2010-10-19

**Authors:** Denis Martinez, Cintia Z Fiori, Diego Baronio, Alicia Carissimi, Renata SR Kaminski, Lenise J Kim, Darlan P Rosa, Ângelo Bos

**Affiliations:** 1 Cardiology Unit, Universidade Federal do Rio Grande do Sul, Brazil, Hospital de Clínicas de Porto Alegre (HCPA), (Ramiro Barcelos, 2350), Porto Alegre, (90035-903), Brazil; 2 Graduate Program in Cardiology and Cardiovascular Sciences, Universidade Federal do Rio Grande do Sul (UFRGS), Cardiology Unit, Hospital de Clínicas de Porto Alegre (HCPA), (Ramiro Barcelos, 2350), Porto Alegre, (90035-903), Brazil; 3 Graduate Program in Medical Sciences, Universidade Federal do Rio Grande do Sul (UFRGS), (Ramiro Barcelos, 2400), Porto Alegre, (90035-903), Brazil; 4 Graduation in Biomedicine, Universidade Federal de Ciências da Saúde de Porto Alegre (UFCSPA), (Sarmento Leite, 245), Porto Alegre, (90050-170), Brazil; 5 Unit of Geriatrics, Hospital São Lucas da PUCRS, (Avenida Ipiranga, 6690), Porto Alegre, (90610-000), Brazil

## Abstract

**Background:**

Intermittent hypoxia (IH), a model of sleep apnea, produces weight loss in animals. We hypothesized that changes in brown adipose tissue (BAT) function are involved in such phenomenon. We investigated the effect of IH, during 35 days, on body weight, brown adipose tissue wet weight (BATww) and total protein concentration (TPC) of BAT.

**Methods:**

We exposed Balb/c mice to 35 days of IH (n = 12) or sham intermittent hypoxia (SIH; n = 12), alternating 30 seconds of progressive hypoxia to a nadir of 6%, followed by 30 seconds of normoxia. During 8 hours, the rodents underwent a total of 480 cycles of hypoxia/reoxygenation, equivalent to an apnea index of 60/hour. BAT was dissected and weighed while wet. Protein was measured using the Lowry protein assay.

**Results:**

Body weight was significantly reduced in animals exposed to IH, at day 35, from 24.4 ± 3.3 to 20.2 ± 2.2 g (p = 0.0004), while in the SIH group it increased from 23.3 ± 3.81 to 24.1 ± 2.96 g (p = 0.23). BATww was also lower in IH than in SIH group (p = 0.00003). TPC of BAT, however, was similar in IH (204.4 ± 44.3 μg/100 μL) and SIH groups (213.2 ± 78.7 μg/100 μL; p = 0.74) and correlated neither with body weight nor with BATww. TPC appeared to be unaffected by exposure to IH also in multivariate analysis, adjusting for body weight and BATww. The correlation between body weight and BATww is significant (rho= 0.63) for the whole sample. When IH and SIH groups are tested separately, the correlations are no longer significant (rho= 0.48 and 0.05, respectively).

**Conclusion:**

IH during 35 days in a mice model of sleep apnea causes weight loss, BATww reduction, and no change in TPC of BATww. The mechanisms of weight loss under IH demands further investigation.

## Background

Obstructive sleep apnea (OSA) [1,2], is characterized by recurrent episodes of partial or total pharyngeal obstruction [3] during sleep, leading to asphyxia. The clinical consequences of OSA emerge from the intermittent hypoxia (IH) and from sleep fragmentation. Overweight is observed in the majority of patients with OSA. The mechanisms of the association between OSA and obesity are still unclear. Excessive fat deposits may participate in OSA as a predisposing factor, as a consequence, or as both [4].

Brown adipose tissue (BAT) function may be involved in obesity [5]. BAT produces heat through the action of uncoupling protein 1 (UCP1) [6]. During exposure to cold, UCP1 is physiologically regulated by catecholamines, thyroid hormones, and leptin [7,8]. BAT function can be stimulated by hypoxia [9]. Contrary to previous concepts [10], BAT is active in 2.5% [11] to 45% [12] of adult humans. BAT activity may need stimulation by cold to be detected [13,14].

Exposure to IH is a model of OSA [15]. Previous reports demonstrated weight loss during exposure to constant hypoxia [16]. Our group reported [17] significant reduction in body weight and in brown adipose tissue wet weight (BATww) in rats subjected to IH for 21 days. OSA patients usually display weight gain [18]. Therefore, the weight loss seen in rodents may represent evidence that obesity is more a predisposing factor than a consequence of OSA.

Because of the effects of hypoxia in several of the mechanisms controlling BAT function, such as, sympathetic function, leptin secretion, thyroid function, we hypothesized that IH can influence the total protein concentration (TPC) in BAT, a surrogate of BAT function. In the present study, we analyzed, in Balb/c mice, the effect of IH during 35 days on body weight, on BATww, and on TPC.

## Methods

Two-month-old male Balb/c mice, from the FEPPS http://www.fepps.rs.gov.br/, Porto Alegre, Brazil, were separated in two groups: 12 mice submitted to 35 days of IH and 12 control mice, submitted to 35 days of sham intermittent hypoxia (SIH). Both groups were housed under temperatures ranging between 22.5 - 24.5°C and received *ad libitum *standard mice chow (Purina-Nutripal, Porto Alegre, RS, Brazil) and water. The protocol was approved by the institutional Ethics Committee and followed the "Guide for the Care and Use of Laboratory Animals" [19]. The mice were weighed at the baseline, 21, and 35 days in a scale with precision of 0.01 g (Marte, model AS 5500C).

**Figure 1:**
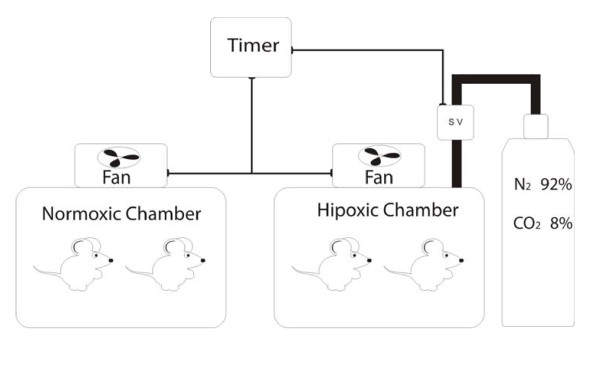
**Diagram of the hypoxic and normoxic chambers**. Solenoid valve (SV).

IH procedures were described in detail before. In brief, during five weeks, 7 days per week, 8 hours a day, from 9 a.m. to 5 p.m., in the lights on period, the animals were placed in the IH system (Figure 1). A mixture with 92% nitrogen and 8% CO_2 _was released in the hypoxia chamber, for 30 seconds. The gas mixture reduced the oxygen fraction from 21% to approximately 7 ± 1% and increased the CO_2 _fraction to approximately 5 ± 1%. Subsequently, a fan insufflated room air into the chamber for 30 seconds, restoring the oxygen fraction to 21%. Each hypoxia/normoxia cycle lasted for 60 seconds; in 8 hours, 480 IH periods occurred, equivalent to an apnea index of 60 per hour. The SIH group remained in an adjacent cage and underwent the same fan activity as the IH group, but no gas mixture entered the cage in the hypoxia cycle. Gas insufflation and fan activity reduced temperature in the hypoxic cage by 0.2 ± 0.3°C, as compared to the normoxic cage that reduced 0.1 ± 0.2°C. Temperature was measured by fast-response thermocouple, averaged from three cage locations. Wind chill effect was minimized by deflectors under the fans.

### Extraction of brown adipose tissue

After 35 days, the animals were anesthetized with ketamine (100 mg/kg) and xylazine (10 mg/kg) intraperitoneally. After deep anesthesia was confirmed, the interscapular BAT was extracted with fine-tipped straight surgical scissors and with anatomical tweezers. BAT was weighed while wet in a digital scale with precision of 0.0001 g (Bel Engineering, Italy) placed in microtubes, frozen in liquid nitrogen, and stored at -80°C until the moment of analysis. After tissue removal, the animals were euthanized by exsanguination under anesthesia.

### Protein determination

The BAT was homogenized and total protein concentration was determined by the Lowry method [20], using as standard a solution of bovine albumin 1 mg/mL. The volumes of the solution used in the calculations were 50, 100 and 150 μL, being represented in a concentration curve. An aliquot of the homogenized BAT (20 μL) was mixed in 780 μL of distilled water and 2.0 mL of reagent C which was prepared with 50 mL of NaHCO3 added with 0.5 mL of reagent B1 (CuSO4. H2O 1%) and 0.5 mL of reagent B2 (sodium tartrate and potassium 2%). After 10 minutes of the addition of reagent C, 0.2 mL of Folin-Ciocalteau reagent, diluted in a proportion of 1:3, was added in distilled water. After 30 minutes, a bluish color was observed and then TPC was measured in a spectrophotometer at 625 nm. The TPC measurement from one mouse in IH group is unavailable because the microtube was lost.

### Statistical Analysis

The results were expressed as mean value and standard deviation. We used SPSS v16 for all statistical analysis (SPSS Chicago, IL). To compare the studied means and variables between two groups we used the Student's t-test for independent samples between two groups, and for more than two groups was used analysis of variance (ANOVA). The significance level for alpha error was p < 0.05. Associations of body weight at day 35 with BATww and TPC levels were examined using Spearman's rank-order correlation coefficients due to the small sample size and the presence of outliers. To adjust correlations for confounders, we utilized linear regression to predict BATww using as regressors: exposure to IH, body weight at day 35, and TPC.

## Results

Data obtained in IH and SIH groups are shown in Table 1 and Table 2. We observed no significant change in body weight in the SIH group whereas in the IH group a significant body weight loss was detected at day 21 and was further intensified at day 35, but non-significantly (p = 0.307). The correlation coefficients of TPC levels against body weight at day 35 and BATww were non-significant.

**Table 1:** Mean and standard deviations of body weight in the two experimental groups

	Sham IH (n = 12)	IH (n = 12)	P between groups
**Body weight at day 1 (g)**	23.3 ± 3.81	24.4 ± 3.33*	0.46

**Body weight at day 21 (g)**	25.8 ± 3.70	21.0 ± 1.71**	**0.0004**

**Body weight at day 35 (g)**	24.1 ± 2.96	20.2 ± 2.20	**0.001**

**P within groups**	0.23	**0.0004**	-

IH: Intermittent hypoxia. * P = 0.0042 comparing with body weight at day 21 and P = 0.0004 comparing with body weight at day 35. ** P = 0.307 comparing with body weight at day 35. Significant P values are in bold type. Body weight compared by repeated measures ANOVA and by independent samples Student's t-test.

**Table 2:** Means and standard deviations of data obtained at day 35 in both experimental groups

	Sham IH (n = 12)	IH (n = 12)	P Value
**Body weight at day 35 (g)**	24.1 ± 2.96	20.2 ± 2.2	**0.001**

**BATww (g)**	0.0372 ± 0.002	0.0318 ± 0.003	**0.00003**

TPC (μg/100 μL)*	213.2 ± 78.7	204.4 ± 44.3*	0.74

IH: Intermittent hypoxia; BATww: Brown adipose tissue wet weight; TPC: Total protein concentration; * n= 11. Significant P values are in bold type.

**Figure 2:**
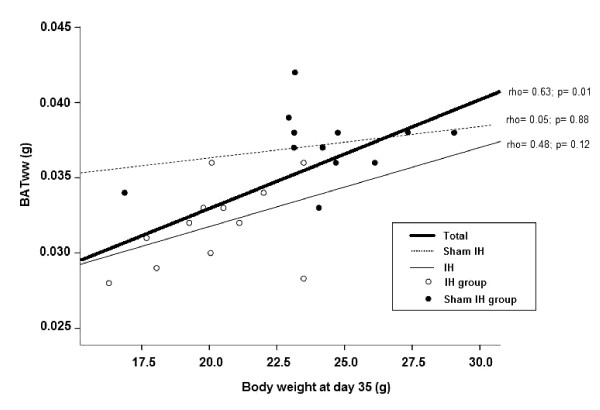
**Scatterplot of brown adipose tissue wet weight by body weight at day 35**.

Significant relationship was observed between body weight at day 35 and BATww for the whole group. Splitting the IH and SIH groups, no significant correlation is seen (Figure 2).

The linear regression model, using BATww as the dependent variable is displayed in Table 3. Exposure to IH was the only variable that remained significant in the model. Partial correlations indicate that exposure to IH was the factor contributing most to the model, followed by TPC and body weight at day 35. Adjusted r square denotes that 61% of the variance in BATww is explained by the three variables.

**Table 3:** Results from linear regression model to predict brown adipose tissue wet weight

		Unstandardized Coefficients		Standardized Coefficient		
**Dependent variable**	**Regressors**	**B**	**Standard error**	**Beta**	**Partial r**	**P value**

**BATww (g)**						
	Intercept	0.027179	0.00648	-	-	0.0004
	IH (exposed, 1)	-0.00419	0.00151	-0.57	-0.54	**0.012**
	TPC (μg/100 μL)	0.000016	0.00002	0.26	0.41	0.067
	Body weight at day 35 (g)	0.000278	0.00027	0.23	0.25	0.2727
	Adjusted R^2 ^= 0.61					

IH: Intermittent hypoxia; BATww: Brown adipose tissue wet weight; TPC: Total protein concentration. Significant P value is in bold type.

## Discussion

The results of the present study indicate that 60 cycles of IH per hour, 8 hours a day, during 35 days cause reduction in body weight and in BATww, but no change in TPC of Balb/c mice. This confirms our previous findings in a rat model of OSA during 21 days [17]. The weight changes are negligible after 21 days.

Body weight at day 35 and BATww are positively correlated. In multivariate analysis, however, the correlation is explained only by exposure to IH, suggesting that IH has direct effect on BATww and that body weight loss is less influent as cause of BATww reduction.

Our finding that weight loss plateaus at 21 days prompts future research comparing BATww at shorter durations of exposure to IH, for instance, one or two weeks. The significant relationship observed between body weight at day 35 and BATww for the whole group could mean that the white and brown fat are simply parts of the total fat deposit which increase and decrease in tandem (Figure 2). The fact, however, that when splitting the IH and SIH groups, a much lower correlation is seen for the SIH group suggest that IH has a direct effect on BATww, supported by the multivariate analysis (Table 3).

BAT activation occurs at room temperatures between 4 - 16°C [9,13,21-23]. In our study, temperature exhibited trivial differences between IH (22.9 ± 2.5°C) and SIH cages (23.2 ± 2.1°C). It is, therefore, improbable that procedure-induced temperature changes could influence BAT behavior.

In conclusion, animals submitted to IH present weight loss and reduction of BATww. We were unable to demonstrate indirect effect of IH on thermogenic activity measuring protein concentration in BAT as surrogate of UCP1 function. Twenty-one days are sufficient to provoke weight loss in IH models. Understanding of the mechanisms of weight loss under IH requires further investigations.

## Abbreviations

HI: Intermittent hypoxia; BAT: Brown adipose tissue; TPC: Total protein concentration; SIH: Sham intermittent hypoxia; BATww: Brown adipose tissue wet weight; OSA: Obstructive sleep apnea; UCP1: Uncoupling protein 1; SV: Solenoid valve.

## Competing interests

The authors declare not having any personal or financial support or involvement with organizations with financial interest in the subject matter or any actual or potential conflict of interest.

## Authors' contributions

DM conceived the study, and participated in its design, coordination and in writing the manuscript; CZF in the design of the study, performed the dissection and weighing of the tissues and the statistical analysis; DB helped to draft the manuscript; RSK participated in the handling of animals; LJK helped to draft the manuscript; AC helped in the statistical analysis; AB helped in the statistical analysis. All authors read and approved the final manuscript.
